# Editorial: Immunomodulatory Roles of Tryptophan Metabolites in Inflammation and Cancer

**DOI:** 10.3389/fimmu.2020.01497

**Published:** 2020-07-16

**Authors:** Juliane Günther, Francesca Fallarino, Dietmar Fuchs, Elisa Wirthgen

**Affiliations:** ^1^Institute of Reproductive Biology, Leibniz Institute for Farm Animal Biology, Dummerstorf, Germany; ^2^Department of Experimental Medicine, University of Perugia, Perugia, Italy; ^3^Institute of Biological Chemistry, Biocenter, Medical University of Innsbruck, Innsbruck, Austria; ^4^Department of Pediatrics, Rostock University Medical Center, Rostock, Germany

**Keywords:** immunomodulation, cancer, inflammation, kynurenine pathway, tryptophan

Tryptophan (TRP) is one of the essential amino acids of mammalian organisms. Besides its primary function in protein synthesis, TRP is metabolized along the serotonin and the kynurenine pathways. During inflammation, the kynurenine pathway (KP) plays a crucial role in the regulation of the immune response, notably as a counter-regulatory mechanism. Three rate-limiting enzymes of KP, tryptophan 2,3-dioxygenase (TDO) and indolamine 2,3-dioxygenase (IDO) 1 and 2, have been described in the literature thus far. The activation of KP results in the generation of a range of biologically active metabolites like kynurenine (KYN), kynurenic acid (KYNA), or quinolinic acid (QUIN). Furthermore, gut microbiota also degrade TRP to bacterial specific metabolites. Both endogenous, as well as bacterial TRP metabolites, have profound effects on host physiology as they contribute to immune homeostasis and impacts on various inflammatory disorders. Moreover, the KP and its metabolites have been linked to tumorigenesis and implicated in several cancers.

Within this Research Topic, 17 articles discuss new and deepen known aspects of the immunomodulatory effects of TRP metabolites in various diseases. New mechanistic insights into the regulation of TRP metabolism and novel signaling pathways activated by specific metabolites are reported. In addition, possible new therapeutic and diagnostic approaches for various diseases, involving the KP, are also described.

During inflammation, IDO-mediated TRP degradation along the KP is strongly induced by pro-inflammatory stimuli. In this issue, Moffett et al. report that the TRP metabolite QUIN, known for its neurotoxic properties, is highly increased in immune cells during inflammatory processes. The authors also propose that during inflammation TRP metabolism may take place mostly in immune cells. This is in contrast to homeostatic conditions and healthy state, where the majority of TRP degradation occurs in the liver. A possible reason for this switch can be explained by two means; first it may be due to an increased NAD^+^ supply, via the KP, required for energy metabolism and redox balance in immune cells, and second, the requirement of KP metabolites for immune regulatory functions. On this regard, Manni et al. investigated the influence of KYN on the regulation of immune responses in a specific mouse model of endotoxin tolerance (ET). The study reports that pharmacologic treatment of dendritic cells (DC) with KYN, prior their activation with lipopolysaccharide (LPS), was able to mimic the effects of repeated stimulation of the same cells with LPS, a process known as ET. Specifically, the authors describe that DC treatment with KYN promotes aryl hydrocarbon receptor (AhR) activation, which then results in the dissociation of the c-SRC kinase from the AhR complex. Activated c-SRC is then responsible for the phosphorylation of the IDO1 enzyme, which in turn acts as important regulator for synthesis of the immunoregulatory cytokine TGF-β. Notably, KYN treated DCs protect mice from lethal endotoxemia *in vivo*. Another inflammatory state in which the TRP metabolism plays a crucial role is the chronic low-grade inflammatory state called inflammaging. In their review, Sorgdrager et al. summarize specific studies highlighting the activation of TRP/KYN pathway in inflammation and discuss the potential role of TRP metabolites as biomarkers in age-related inflammatory diseases. Furthermore, the immunomodulatory role of kynurenines in various infectious and non-infectious diseases and genetic manipulation of the major enzymes involved in their production is reviewed in detail by Boros and Vécsei. The work by Costantini et al. describes an additional level of complexity in the immunoregulatory function exerted by TRP metabolites produced by microbes. Those by utilizing TRP via alternative pathways produce selective metabolites including indoles or tryptamine, thus, cross-regulating microbes and host metabolism, resulting in either commensalism or pathogenic effects. Specifically, some of these metabolites exert direct effects on immune cells as they function as ligands of specific xenobiotic receptors, such as, AhR. The activation of these receptors results in the transcription of a variety of genes associated with immune control, gut-homeostasis as well as drug metabolism. In addition to bacteria, TRP metabolism has been also described in opportunistic or pathogenic fungi. By this means, fungi are able to modulate Th17 response by the release of TRP metabolites, thus also inducing IDO1 in specific immune cells.

Dysregulation of TRP metabolism has been described in various inflammatory/autoimmune disorders. In particular, IDO1 activation and production of KP metabolites in peripheral and CNS disorders was reviewed by Huang et al.. Findings in peripheral disorders, including rheumatoid arthritis or atherosclerosis, suggest that IDO1 activation mediates overall anti-inflammatory activity, exerting a protective function limiting disease severity. Accordingly, epigenetic regulation of IDO1 affects the susceptibility to arthritis and other inflammatory disorders. An anti-inflammatory activity of kynurenines was also discussed in CNS disorders such as, Huntington's disease, Alzheimer's disease, and multiple sclerosis (MS). The study by Gaetani et al. confirmed that MS is characterized by misbalances in TRP metabolism, suggesting TRP metabolites as biomarker in this disease. Specifically, they found urinary KYN, as well as the KYN/TRP ratio reduced in MS patients, indicating a decreased degradation of TRP via the KP. However, other studies that had determined TRP metabolites in serum instead of urine describe an activation of KP in the periphery of MS patients. In the present study, the authors further found an increase of the microbiota-derived TRP metabolite indole-3-propionic acid in the urine of MS patients. This could be a compensation to counteract the adverse effects of acute inflammation. An altered microbiota-mediated TRP metabolism was also described in obesity by Cussotto et al.. Specifically, in obese patients, systemic inflammation, reflected by increased plasma levels of C-reactive protein, IL-6 and KYN/TRP ratio, was associated with a reduced production of microbiota-derived indoles, indicating changes in microbiota composition and function. Lower production of indoles may affect innate and adaptive immune responses, as they function as ligands of the AhR, which mediates regulatory/anti-inflammatory functions and prevents tissue damage. Accordingly, a diminished activation of AhR by reduced indoles, may contribute to a disregulation of gut immune homeostasis in obesity. The connection between peripheral inflammation and psychiatric disorders has been demonstrated by De Picker et al.. They found an increase in plasma markers for acute inflammation associated to a reduction of both the anti-inflammatory metabolite KYNA and the KYNA/KYN ratio in patients with acute psychotic illness. This decrease may be indicative of an increased transfer of TRP or KYN through the blood-brain barrier and the CNS, serving as a substrate for local synthesis of KYNA in brain tissue.

Due to their immunoregulatory properties, TRP metabolites, generated through the KP, have also been linked to tumorigenesis. In this context, a strong expression of TRP-metabolizing enzymes by cancer cells promotes the establishment of an immunosuppressive microenvironment resulting in impaired immune response against tumor cells. On this regard, the work of Nafia et al. shows that in a mouse sarcoma model a combined application of the IDO1 inhibitor GDC-0919 with an anti-PDL1 antibody leads to a transient decrease in the plasma KYN levels. However, the treatment did not show any anti-tumoral activity and did not affect tumor immune cell infiltrate. Notably, transcriptome analyses of the tumor indicated that the IDO1 inhibitor either as single agent or combined with anti-PDL1 significantly induced a downregulation of the expression of several genes required for natural killer effector function. In another study, Riess et al. demonstrated that the inhibitor of cyclin-dependent kinases Dinaciclib inhibited IFN-γ induced synthesis of KP metabolites in glioblastoma cell lines. In contrast, the non-targeted conventional chemotherapeutic drug Temozolomid tended to activate the KP, which may represent an adverse effect inducing tumor immune escape. These data point out the limitations of certain conventional anti-tumor therapies and highlight the potential of targeted therapies. The influence of TRP metabolism on cancer comorbidities such as anemia, fatique, and depression is discussed by Lanser et al.. They reviewed results on how various clinical and lifestyle interventions, like nutrition and physical activity, could influence TRP breakdown and thus possibly anemia and depression. In contrast, a further review article by Günther et al. discuss the limitations of cancer therapy with IDO inhibitors due to pharmacokinetic features as well as side- and off-target effects. The described effects should be taken into account when classifying controversial results regarding the efficacy of IDO1 inhibitors leading to IDO1 inhibition and cancer treatment. However, the authors also highlight the potential of these inhibitors independent of the IDO1 signaling pathway.

Cancer cells are also a widely used model for mechanistic studies of the TRP metabolism pathways and their regulation. Mohapatra et al. show that in glioblastoma cell (GC) lines under hypoxic conditions the Hypoxia-inducible factor 1-alpha, HIF1α mediates TDO2 downregulation both at mRNA and protein level. The downregulation of TDO2 through hypoxia inhibits unnecessary consumption of the essential amino acid TRP and is reversible as re-oxygenation rescued TDO2 expression. These data suggest that the regulation of TDO2 expression by HIF1α may be involved in modulating anti-tumor immunity in GCs. Another way of regulating TDO2 in GCs at the transcriptional level was investigated by Kudo et al.. They found that the LAP form of the transcription factor CEBPβ is necessary for the expression of TDO2 in these cancer cells. Moreover, the activation of this transcription factor seems to be IL-1β-dependent. In cancers, it has been shown that an increased activity of the rate-limiting enzymes TDO2 or IDO1 promotes the development of an immunosuppressive microenvironment promoting tumor immune escape. Thus, several enzyme-inhibitors are currently tested *in vitro* as well as in clinical trials. In this context, Wirthgen et al. show that the IDO inhibitor 1-methyltryptophan (1-MT) drives the KP toward the KYNA branch in mice as well as in humans. Interestingly, similar results in IDO^−/−^ mice indicate that this effect seems not to be mediated by IDO1. Under inflammatory conditions, KYNA mediates mainly immunosuppressive effects as shown in various experimental *in vitro* and *in vivo* models. The increase of KYNA may represent one potential way of action of 1-MT and should be considered for preclinical studies and therapeutic applications in humans. On this regard, potential mechanisms and anti-inflammatory functions of KYNA were also investigated by Mándi et al. using a monocytic cancer cell line. The authors show that the increase of the TNFα-stimulated Gene-6 expression by KYNA and in particular by new KYNA analogs, may represent one of the mechanisms explaining their immunosuppressive effects, as a feedback mechanism on TNFα production.

Constant increasing research activities reveal new insights into the importance of TRP metabolites for inflammation and also make them interesting as biomarkers. We anticipate that these studies will provide state-of-the-art information about immunomodulatory roles of TRP metabolites and open additional areas for future investigation.

## Author Contributions

All authors listed have made a substantial, direct and intellectual contribution to the work, and approved it for publication.

## Conflict of Interest

The authors declare that the research was conducted in the absence of any commercial or financial relationships that could be construed as a potential conflict of interest.

